# Effects of inoculation by arbuscular mycorrhizal fungi on the composition of the essential oil, plant growth, and lipoxygenase activity of *Piper aduncum* L.

**DOI:** 10.1186/s13568-019-0756-y

**Published:** 2019-02-26

**Authors:** Joyce Solange F. de Oliveira, Luciana P. Xavier, Alba Lins, Eloisa Helena A. Andrade, José Guilherme S. Maia, Andréa H. de Mello, William N. Setzer, Alessandra R. Ramos, Joyce Kelly R. da Silva

**Affiliations:** 1 Programa de Pós-Graduação em Biotecnologia, Universidade Federal do Pará, Belém, Pará Brazil; 2 Coordenação de Botânica, Museu Paraense Emílio Goeldi, Belém, Pará Brazil; 3 Programa de Pós-Graduação em Recursos Naturais da Amazônia, Universidade Federal do Oeste do Pará, Santarém, Pará Brazil; 4 Instituto de Estudo do Desenvolvimento Agrário e Regional, Universidade Federal do Sul e Sudeste do Pará, Marabá, Pará Brazil; 5 Department of Chemistry, University of Alabama in Huntsville, Huntsville, AL 35899 USA; 6 Instituto de Estudos em Saúde e Biológicas, Universidade Federal do Sul e Sudeste do Pará, Marabá, Pará Brazil

**Keywords:** Arbuscular mycorrhizal fungi, Volatile compounds, Dillapiole, Lipoxygenase, Secondary metabolites

## Abstract

The aim of this study was to evaluate the changes in the production of secondary metabolites *Piper aduncum* seedlings were inoculated by spores of the arbuscular mycorrhizal fungi (AMF) *Rhizophagus clarus* and *Claroideoglomus etunicatum. P. aduncum* seedlings were inoculated by spores of *R. clarus* and *C. etunicatum* and then, development parameters, root colonization, lipoxygenase (LOX) activity, and essential oil (OE) chemical composition were monitored at 30, 60 and 90 days’ post-inoculation (dpi). The inoculation had influenced the plant height and root length at 30 and 90 dpi and microscopic analysis of roots showed the presence of hyphae, arbuscules and vesicles in the inoculated plants. Phenylpropanoids and sesquiterpene hydrocarbons were the main compounds in the EO. In the leaves, the concentration of phenylpropanoids showed a decrease, mainly at 60 dpi, with increased sesquiterpene hydrocarbon production. The main compounds were dillapiole, myristicin, and germacrene D; the dillapiole concentration decreased in all treatments. LOX activity had an increase in the leaves and roots at 90 dpi. These results suggest that alterations in the secondary metabolites of *P*. *aduncum* can be induced by its mechanisms of resistance during AMF interaction.

## Introduction

*Piperaceae* have wide distributions in tropical and subtropical regions, and are known as a pantropical family with approximately 2700 species mainly of the genus *Piper* (The Plant List 2013). *Piper aduncum* L. is a bush native to tropical regions of the Americas, but it was introduced to Asia during the nineteenth century (Hartemink 2001; Yuncker 1972). In the Amazon region, it is commonly known as “pimenta-de-macaco”, and used in popular medicine to treat intestinal apathy and stomach problems (Sousa et al. 2008). In addition, the *P*. *aduncum* essential oil (EO) has demonstrated several biological properties, such as antifungal (Guerrini et al. 2009), antimicrobial (Kloucek et al. 2005), insecticidal (Misni et al. 2011), and larvicidal (Almeida et al. 2009) activities. These biological properties can be attributed to its main compound, dillapiole, a phenylpropanoid which presents in amounts of 31.5 to 91.1% (Maia et al. 1998).

The biosynthesis of secondary metabolites in medicinal and aromatic plants depends on genetic, physiological, and environmental factors (Freitas et al. 2004). Among these factors, the symbiotic association of plants by root colonization by arbuscular mycorrhizal fungi (AMF) can produce a difference in its biosynthesis of secondary metabolites (Carlsen et al. 2008). AMF belongs to the *Glomeromycota phylum* and the *Acaulospora*, *Entrophospora*, *Gigaspora*, *Glomus*, *Sclerocystis* and *Scutellispora* genera (Oehl et al. 2011). AMFs have shown associations with about 80% of ground plants in natural ecosystems and cultivated agroecosystems, varying the colonization level according to plant genotype (Bonfante and Genre 2010; Smith and Read 2008).

The plants colonized by AMFs are more tolerant to low availability of water in the soil, making more efficient use of the absorbed water. In addition, they improve the plant nutrition, development, and the content of the essential oils, due to changes in the biosynthesis of secondary metabolites (Al-karaki et al. 2004; Nell et al. 2010). Thus, the aim of this study was to evaluate the changes in the production of secondary metabolites during the association of *P. aduncum* with AMFs.

## Materials and methods

### Plant material and cultivation

*P. aduncum* was collected in Belém/PA, Brazil, and a voucher specimen was deposited under register MG 218522 in the Emílio Goeldi Museum herbarium, city of Belém, Pará, Brazil. Cuttings containing 1 to 2 nodes were propagated and conditioned in vermiculite expanded type B substrate (Urimamã Mineração Ltda, Santa Maria da Boa Vista, Brazil), and kept in a greenhouse under 70% shading. The commercial nutrient solution (Biofert Root) was applied to promote root development and reapplied after 15 days, and the cuttings were moistened daily. After 21 days, the roots had developed, and seedlings were transplanted into polypropylene bags of approximately 9 cm in diameter, on a commercial substrate containing a mixture of limestone, castor oil, bone meal, and expanded vermiculite type B.

### Multiplication of AMF spores and production of fungal inoculant

AMF spores (*Rhizophagus clarus* and *Claroideoglomus etunicatum*) were obtained from rhizosphere soil samples from the southeast Pará State, Amazon region (Brazil). They were multiplied in a greenhouse in sterile sand, using *Brachiaria brizantha* as trap culture (Da Luz et al. 2016). The identification of species was realized by morphological comparison based in the International Culture Collection of (Vesicular) Arbuscular Mycorrhizal Fungi (INVAM 1992). Inoculates, with the proportion of 50% each fungal species, composed of a mixture of spores (density of 90 spores/g soil), hyphae, root fragments and sterile sand, were used during the inoculation. Holes with approximately 2 cm deep were opened and the 6 g of inoculum was surface-spread on the roots. Non-inoculated seedlings were used as the control group.

### Experimental design

Experimental design was performed in completely randomized blocks. Each group was composed of 10 plants, which were labeled as control (non-inoculated) and AMF (inoculated by AMFs). Roots and leaves were collected at 30, 60 and 90 days post inoculation (dpi) to monitor the mycorrhizal colonization, plant development, secondary metabolites, and LOX activity. All analyzes were performed in biological triplicates.

### Mycorrhizal colonization in *P. aduncum* roots

For the visualization of mycorrhizal colonization, usual techniques in plant anatomy were employed (Kraus and Arduin 1997). Root fragments of approximately 1 cm were fixed during 24 h in glutaraldehyde 1% in 0.1 M phosphate buffer, pH 7.2 (according to Karnovsky 1965, with modifications). Afterward, the samples were dehydrated with a series of butyl alcohol treatments and then encased in histological paraffin (Johansen 1940). Longitudinal sections (12 μm thick) were obtained using an automatic microtome (Leica^®^ RM 2245, Nussloch, Germany), the sections were stained with safranine and astra blue (Gerlach 1969), and mounted in Entellan^®^ synthetic resin (Merck, Darmstadt, Germany). Photomicrographs were obtained using in Cannon digital camera (model A65015), coupled to a Zeiss microscope (model 426126.)

### Plant development evaluation

The developmental parameters evaluated were: plant height (cm), number of leaves, plant basal stem (mm), number of nodes, root length (cm), and the fresh mass of leaves and roots (g) for each plant per replicate. The fresh leaf biomass production was based in the total weight per plant and the fresh root biomass in the total weight of the roots per plant.

### Extraction and analysis of the essential oils

The essential oil fractions from fresh leaves and roots (2.0 g) of *P. aduncum* were obtained by simultaneous distillation–extraction process using a Likens-Nickerson apparatus for 2 h and *n*-pentane (3 mL) as solvent. After extraction, an aliquot (1.0 μL) of the organic phase was analyzed by gas chromatography. Qualitative analysis was carried out on a GC–MS (Shimadzu QP2010 plus instrument) under the following conditions: Rtx-5MS silica capillary column (30 m × 0.25 mm × 0.25 mm film thickness); programmed temperature, 60–240 °C (3 °C/min); injector temperature, 200 °C; carrier gas, helium, adjusted to a linear velocity of 1.2 mL/min; injection type, splitless; split flow was adjusted to yield a 20:1 ratio; septum sweep was a constant 10 mL/min; EIMS, electron energy, 70 eV; temperature of the ion source and connection parts, 200 °C. The retention index was calculated for all the volatile constituents using a homologous series of *n*-alkanes (C8–C32, Sigma-Aldrich) (Van Den Dool and Kratz 1966). The identification of compounds was performed by comparison of mass spectrum and retention index with data present in the libraries of Adams (2007) and NIST (2011).

### In vitro lipoxygenase (LOX) activity

The substrate was prepared using 78 μL of linoleic acid (Sigma-Aldrich, USA) and 90 μL Tween 20 (Sigma-Aldrich), mixed with 10 mL of boiling water and a few drops of sodium hydroxide (0.5 N). The final volume was adjusted to 25 mL, resulting in a sodium linoleate solution (10 mM), which was stored at − 20 °C. The LOX activity determination was carried out with 5 μL of crude leaf extract and 50 μL of sodium linoleate (10 mM), mixed with 1950 μL of sodium phosphate buffer (50 mM) at pH 6.5. The absorbance at 234 nm for the reaction was monitored for 60 s, using a UV–Visible spectrophotometer (Meireles et al. 2016).

### Statistical analysis

All analyses were compared with the control group and the data were expressed as mean ± standard deviation. Analyses of variance were conducted using GraphPad 6.0, followed Bonferroni tests whenever appropriate. Differences at *p *< 0.05 were considered statistically significant.

## Results

### Monitoring of colonization of *P. aduncum* roots by AMFs

**Fig. 1:**
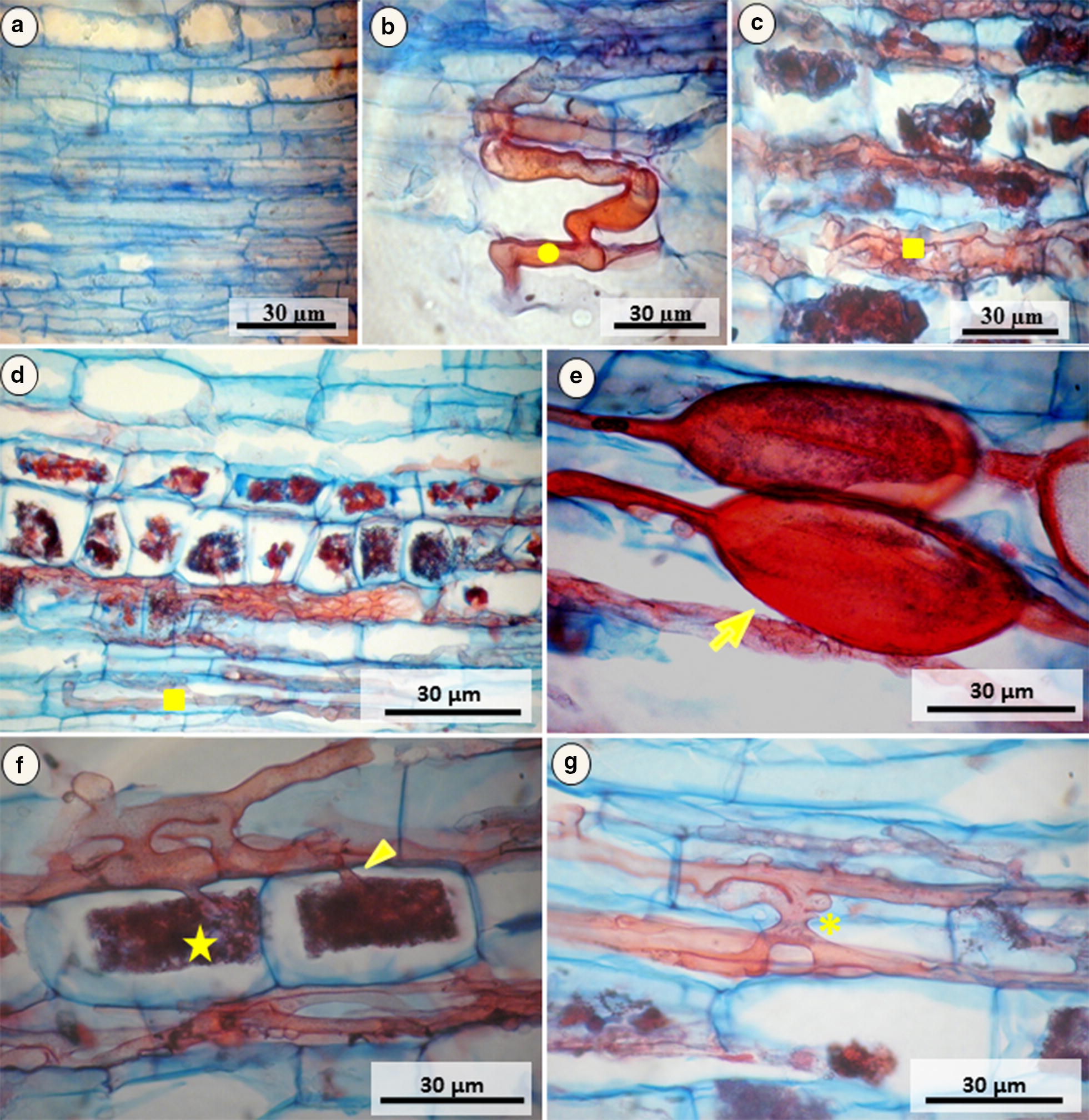
Longitudinal Section of *Piper aduncum* root inoculated with mycorrhizal fungi arbuscular (AMFs). **a** Control sample; **b** Penetration of hyphae in epidermal cells with formation of hyphopodium in 30 dpi; **c** Intense colonization in 60 dpi; **d** Colonization in 90 dpi; **e** Formation of vesicles in 90 dpi; **f** Hyphae inter- and intracellularly and formation of arbuscules in 90 dpi; **g** Hepatic anastomosis in 90 dpi; (Filled circle) Hyphopodium; (Filled square) Intracellular hyphae; (  ) Vesicles; (★) Arbuscules; (Filled triangle) Arbuscular trunks; (  ) Hyphatic anastomosis

Histological sections of *P. aduncum* roots inoculated by AMF revealed evidence of the presence of mycorrhizal structures such as hyphae, arbuscules, and vesicles, which were absent in the control plants (Fig. 1a). At 30 days post inoculation (dpi), the cortex was colonized, and the presence of penetration apparatus composed by hyphopodium and hyphae (Fig. 1b) were observed. At 60 dpi, an intense colonization was observed in the radicular cortex with presence of numerous intracellular hyphae (Fig. 1c). At 90 dpi, the colonization showed completely establishment due to the presence of several hyphae, arbuscles and vesicles (Fig. 1d–f). In addition, hyphatic anastomosis was also observed (Fig. 1g).

### Growth and development of inoculated and non-inoculated plants

Inoculation effects were evaluated on development of *P. aduncum* plants and the values for each parameter were compared to the control group (Table 1). Statistical differences were not observed for parameters such as basal stem diameter and leaf numbers between inoculated plants and control group (Table 1). Inoculated plants displayed a gradual increase in plant height at 30, and 90 dpi. The number of nodes in inoculated plants was higher at 90 dpi but had not displayed statistical differences at 30, and 60 dpi. These results indicate that inoculation benefits were demonstrated after 90 dpi. The increase of fresh mass was observed only in the roots at 30 dpi.

**Table 1:** Developmental parameters of *P. aduncum* during inoculation by AMFs

dpi	Treatments	Evaluation parameters^a^						
		Basal stem (mm)	Leaves	Node	Height (cm)	Root (cm)	Fresh weight (leaves)	Fresh weight (root)
30	Control	3.9 ± 0.0	4.7 ± 0.6	4.3 ± 0.6	21.0 ± 1.7	37.7 ± 1.2	4.9 ± 0.9	4.3 ± 0.7
	AMF	3.8 ± 0.2	5.3 ± 0.6	5.3 ± 0.6	30.5 ± 2.2*	44.0 ± 1.7	6.9 ± 1.1	7.5 ± 0.9*
60	Control	5.5 ± 0.9	7.3 ± 0.6	7.0 ± 1.0	37.7 ± 2.5	36.4 ± 1.1	2.2 ± 0.8	3.8 ± 0.9
	AMF	6.3 ± 0.4	6.7 ± 0.6	6.7 ± 0.6	41.3 ± 1.5	30.1 ± 7.0	4.5 ± 0.2	5.2 ± 1.2
90	Control	6.1 ± 0.9	15.0 ± 1.0	16.0 ± 0.8	53.8 ± 0.9	53.3 ± 0.5	14.5 ± 1.9	7.8 ± 0.8
	AMF	7.2 ± 0.7	16.3 ± 2.1	18.3 ± 0.6*	61.1 ± 4.3*	57.0 ± 1.0	17.2 ± 1.5	8.8 ± 0.4

dpi: Days post inoculation; Control: *P. aduncum* non-inoculated with AMF; AMF: *P. aduncum* inoculated with AMF

* Statistical difference according to Bonferroni-test (*p *< 0.05)

^a^Mean ± standard deviation (n = 3)

### Variation of volatile compounds in the leaves and roots during the colonization by AMF

The GC–MS analysis of volatiles of *P. aduncum* leaves and roots resulted in the identification of 65 and 79 compounds, respectively. The most representative compound classes identified were phenylpropanoids and sesquiterpene hydrocarbons such as dillapiole, myristicin, germacrene D and elemicin. In the leaves, the phenylpropanoid concentrations displayed a difference between inoculated and non-inoculated plants at 60 dpi (Table 2). The main change was observed at 60 dpi, with a drastic decrease (87.94–52.58%) in inoculated plants. In the roots, the most representative classes were phenylpropanoids (≈ 95%) and sesquiterpene hydrocarbons (≈ 12%). The production of phenylpropanoid showed an increase only at 30 dpi. However, concentrations of sesquiterpene hydrocarbons were lower in the inoculated plants at 30 and 60 dpi (Table 2).

**Table 2:** Comparison of volatile components produced in inoculated and non-inoculated leaves of *P. aduncum* (Mean standard deviation)

Compound	RI^calc^	RI^lit^	30 dpi^a^		60 dpi^a^		90 dpi^a^	
			Control	AMF	Control	AMF	Control	AMF
α-Thujene	931	924				0.33 ± 0.47	0.48 ± 0.67	0.63 ± 0.35
β-Pinene	976	974				0.21 ± 0.30	0.15 ± 0.21	0.22 ± 0.23
Sabinene	977	969				0.24 ± 0.34	0.79 ± 0.53	0.84 ± 0.03
α-Phellandrene	996	1002					0.14 ± 0.20	0.18 ± 0.01
δ-2-Carene	1006	1001						0.08 ± 0.01
δ-3-Carene	1015	1008					0.06 ± 0.08	
Limonene	1028	1024				0.32 ± 0.33	0.21 ± 0.29	0.33 ± 0.29
(*Z*)-β-Ocimene	1032	1032		0.02 ± 0.00		1.10 ± 0.21	2.07 ± 0.33	2.59 ± 0.02
(*E*)-β-Ocimene	1043	1044		0.12 ± 0.00		2.84 ± 0.01	6.58 ± 2.03	6.76 ± 0.41
γ-Terpinene	1046	1054				0.27 ± 0.38	0.21 ± 0.05	0.41 ± 0.21
Terpinolene	1084	1086						0.34 ± 0.00
*allo*-Ocimene	1126	1128				0.62 ± 0.03		1.64 ± 0.16
Terpinen-4-ol	1179	1174		0.11 ± 0.24		0.36 ± 0.30	0.32 ± 0.24	0.56 ± 0.27
Piperitone	1246	1249	0.04 ± 0.05	0.43 ± 0.92	0.16 ± 0.23	1.40 ± 1.00	1.12 ± 0.75	1.38 ± 1.23
α-Terpinyl formate	1252	1306						0.41 ± 0.07
Safrole	1282	1285	0.01 ± 0.01		0.04 ± 0.01	0.19 ± 0.27	0.04 ± 0.06	
δ-Elemene	1324	1335	0.08 ± 0.06	0.28 ± 0.28	0.54 ± 0.07	1.66 ± 0.08	2.33 ± 0.49	2.38 ± 1.07
α-Cubebene	1344	1345				0.05 ± 0.01		0.04 ± 0.04
α-Ylangene	1366	1373		0.04 ± 0.05	0.05 ± 0.06	0.59 ± 0.13	0.38 ± 0.06	0.54 ± 0.17
α-Copaene	1368	1374		0.25 ± 0.28	0.17 ± 0.19	1.13 ± 0.27	0.40 ± 0.57	0.99 ± 0.01
β-Elemene	1382	1389	0.14 ± 0.08	0.35 ± 0.16	0.73 ± 0.01	1.92 ± 0.36	1.71 ± 0.04	2.06 ± 0.59
*n*-Tetradecane	1399	1400				0.05 ± 0.01		0.02 ± 0.01
β-Caryophyllene	1412	1417	0.27 ± 0.05	2.62 ± 1.03	2.16 ± 0.95	5.58 ± 0.76	5.00 ± 0.18	4.84 ± 0.54
γ-Elemene	1422	1434	0.23 ± 0.16	0.50 ± 0.24	0.45 ± 0.63	2.18 ± 0.58	2.07 ± 0.08	2.23 ± 0.64
β-Copaene	1430	1430			0.42 ± 0.59	0.05 ± 0.01		0.03 ± 0.04
Aromadendrene	1435	1439				0.13 ± 0.08	0.05 ± 0.01	0.10 ± 0.03
6,9-Guaiadiene	1438	1442				0.11 ± 0.03	0.07 ± 0.01	0.11 ± 0.05
Isogermacrene D	1441	1445		0.01 ± 0.03	0.07 ± 0.00	0.35 ± 0.06	0.29 ± 0.01	0.40 ± 0.13
α-Humulene	1452	1452	0.05 ± 0.07	0.62 ± 0.47	0.72 ± 0.31	2.38 ± 0.11	1.50 ± 0.18	1.73 ± 0.24
*allo*-Aromadendrene	1456	1458		0.01 ± 0.03		0.29 ± 0.11	0.19 ± 0.07	0.24 ± 0.01
Dauca-5,8-diene	1469	1471		0.02 ± 0.04		0.07 ± 0.01		0.06 ± 0.04
γ-Muurolene	1472	1478				0.26 ± 0.04	0.13 ± 0.01	0.17 ± 0.12
Germacrene D	1474	1484	1.28 ± 0.78	2.78 ± 0.94	3.19 ± 0.08	5.49 ± 1.32	6.50 ± 0.45	3.84 ± 0.73
β-Selinene	1485	1489		0.02 ± 0.04		1.33 ± 1.34	0.07 ± 0.10	0.20 ± 0.06
α-Selinene	1485	1498			0.02 ± 0.02			
Viridiflorene	1488	1496				0.17 ± 0.02	0.14 ± 0.09	0.19 ± 0.16
Bicyclogermacrene	1492	1500	0.23 ± 0.18	0.36 ± 0.40	0.70 ± 0.04	2.15 ± 0.34	2.56 ± 0.59	2.74 ± 0.69
α-Muurolene	1495	1500		0.06 ± 0.08	0.14 ± 0.00	0.59 ± 0.11	0.24 ± 0.33	0.61 ± 0.21
*n*-Pentadecane	1496	1500	0.78 ± 0.29	2.28 ± 0.46	1.66 ± 1.07	3.77 ± 0.95	2.48 ± 0.23	
(*E*,*E*)-α-Farnesene	1502	1505		0.05 ± 0.11	0.07 ± 0.04	0.50 ± 0.18	0.17 ± 0.07	0.19 ± 0.01
γ-Cadinene	1509	1513	0.01 ± 0.01	0.02 ± 0.04	0.06 ± 0.04	0.32 ± 0.06	0.16 ± 0.02	0.54 ± 0.08
δ-Cadinene	1514	1522	0.07 ± 0.03	0.31 ± 0.21	0.19 ± 0.02	0.85 ± 0.05	0.53 ± 0.14	0.57 ± 0.42
Myristicin	1517	1517	1.53 ± 0.41	0.61 ± 0.60	2.71 ± 1.11	4.70 ± 0.11	3.26 ± 0.88	2.41 ± 2.32
7-*epi*-α-Selinene	1526	1520						0.01 ± 0.01
*trans*-Cadina-1.4-diene	1529	1533				0.11 ± 0.02	0.04 ± 0.00	0.05 ± 0.04
α-Cadinene	1533	1537				0.06 ± 0.01		0.06 ± 0.02
α-Calacorene	1538	1544		0.01 ± 0.03		0.22 ± 0.13	0.05 ± 0.06	0.11 ± 0.06
Elemol	1542	1548						0.07 ± 0.14
Germacrene B	1554	1559				0.07 ± 0.01	0.05 ± 0.01	0.08 ± 0.08
(*E*)-Nerolidol	1559	1561			0.08 ± 0.11	0.41 ± 0.10	0.17 ± 0.01	0.34 ± 0.08
Palustrol	1566	1567				0.04 ± 0.05	0.02 ± 0.03	0.06 ± 0.04
Spathulenol	1573	1577			0.12 ± 0.16	0.40 ± 0.57		0.23 ± 0.00
Caryophyllene oxide	1578	1582	0.065 ± 0.09	0.05 ± 0.10	0.12 ± 0.06	0.79 ± 0.48	0.27 ± 0.19	0.30 ± 0.42
Globulol	1582	1590				0.09 ± 0.05		0.11 ± 0.04
Viridiflorol	1591	1592				0.23 ± 0.12	0.50 ± 0.59	0.42 ± 0.46
Humulene epoxide II	1606	1608	0.04 ± 0.06	0.04 ± 0.09	0.01 ± 0.01	0.08 ± 0.11	0.03 ± 0.04	0.20 ± 0.21
1,10-di-*epi*-Cubenol	1609	1618						0.04 ± 0.03
Dillapiole	1623	1620	93.74 ± 0.01	86.11 ± 9.45	83.57 ± 3.39	44.73 ± 1.67	48.66 ± 2.11	39.36 ± 6.58
*epi*-α-Muurolol	1644	1640			0.02 ± 0.02		0.16 ± 0.11	0.58 ± 0.30
α-Muurolol	1646	1644				0.25 ± 0.35	0.02 ± 0.03	0.07 ± 0.05
α-Cadinol	1654	1652			0.09 ± 0.13		0.36 ± 0.08	0.44 ± 0.52
Apiole	1669	1677	0.42 ± 0.26	0.11 ± 0.06	1.24 ± 0.28	2.01 ± 2.84	2.54 ± 0.92	1.83 ± 1.43
*n*-Tetradecanol	1676	1671		1.54 ± 3.23	0.02 ± 0.02	0.01 ± 0.13	0.10 ± 0.01	0.12 ± 0.01
Monoterpene hydrocarbons				0.14 ± 0.00		5.93 ± 2.07	10.69 ± 4.39	14.02 ± 1.72
Oxygenated monoterpenes			0.04 ± 0.05	0.54 ± 1.16	0.16 ± 0.23	1.76 ± 1.30	1.44 ± 0.99	2.35 ± 1.57
Sesquiterpene hydrocarbons			3.14 ± 1.71	10.59 ± 4.29	11.34 ± 4.12	32.49 ± 7.20*	27.17 ± 3.88	25.22 ± 6.38
Oxygenated sesquiterpenes			0.11 ± 0.15	0.09 ± 0.19	0.44 ± 0.49	2.29 ± 1.83	1.53 ± 1.08	2.86 ± 2.29
Phenylpropanoids			95.86 ± 0.92	86.83 ± 10.11	87.94 ± 4.85	52.58 ± 5.02*	55.16 ± 4.03	44.26 ± 10.57
Others				1.54 ± 3.23	0.02 ± 0.02	0.01 ± 0.13	0.10 ± 0.01	0.12 ± 0.01
Total			99.15 ± 2.83	99.73 ± 18.98	99.90 ± 9.71	95.06 ± 17.55	96.09 ± 14.38	88.83 ± 22.54

dpi: Days post inoculation; Control: *P. aduncum* non-inoculated with AMF; AMF: *P. aduncum* inoculated with AMF; RI cal: Retention index calculated; RI lit: Retention Index of Library

* Statistical difference according to Bonferroni test (*p* < 0.05)

^a^Mean ± standard deviation (n = 3)

**Fig. 2:**
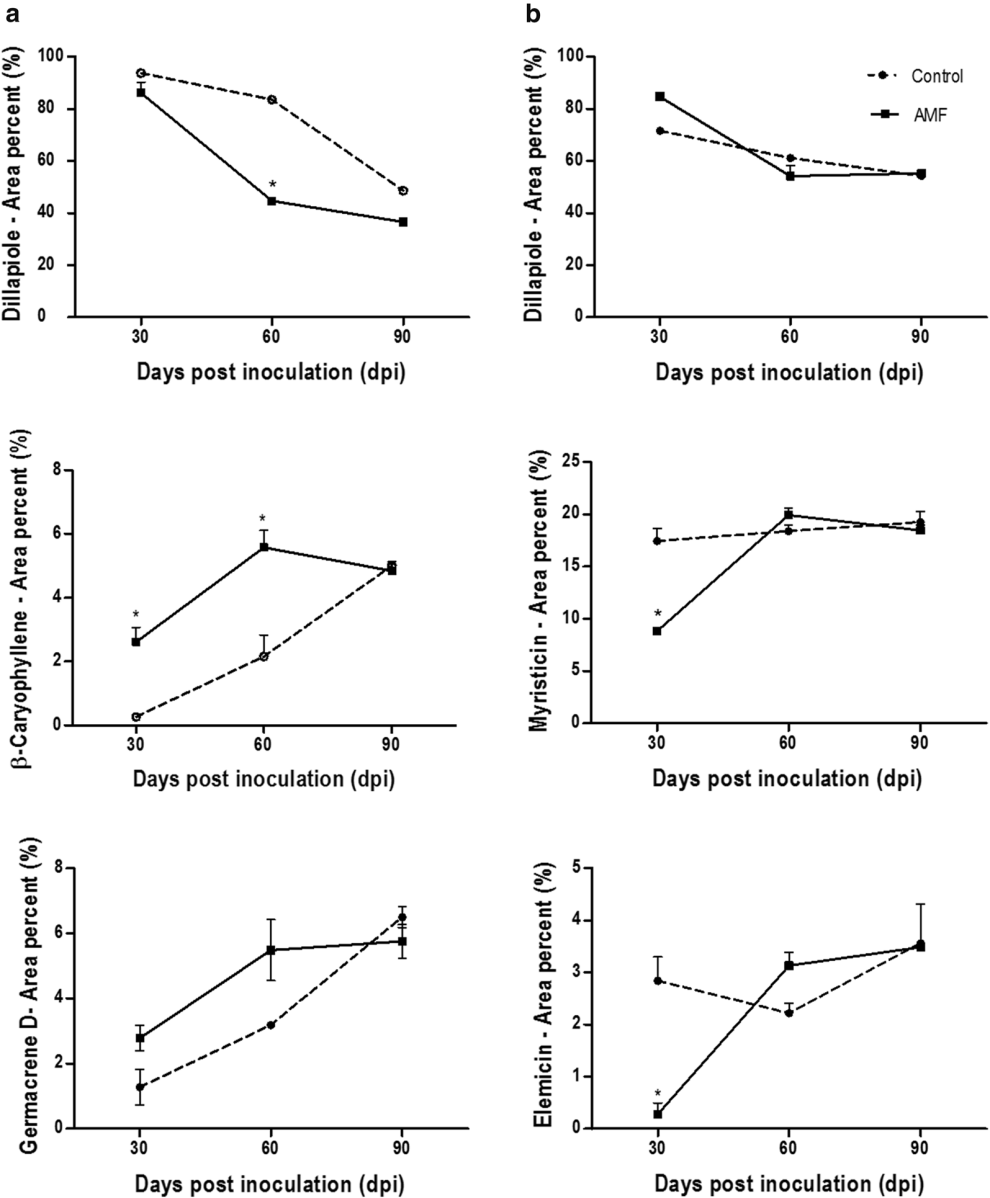
Major compounds produced by *P. aduncum* during inoculation with arbuscular mycorrhizal fungi (AMFs). **a** Leaves; **b** Roots. *Statistical difference according to Bonferroni test (*p* < 0.05)

At 30 dpi, quantitative and qualitative changes were observed in the leaves. Quantitatively, a decrease in the dillapiole content (93.74–86.11%), and an increase of β-caryophyllene (0.27–2.62%) and germacrene D production (1.28-2.78%) was observed (Fig. 2). Qualitatively, the inoculated plants produced additional compounds not observed in the control plants, including (*E*)-β-ocimene (0.12%), terpinen-4-ol (0.11%), α-copaene (0.25%) and *n*-tetradecanol (1.54%). At 60 dpi, there was an increase in the contents of β-caryophyllene (2.16–5.58%), germacrene D (3.19–5.49%), myristicin (2.71-4.70%), and a greater decrease of dillapiole (83.57–44.73%). Several monoterpenes and sesquiterpenes were produced only by inoculated plants, such as (*Z*)-β-ocimene (1.10%), (*E*)-β-ocimene (2.84%), and β-selinene (1.33%). At 90 dpi, only inoculated plants produced the monoterpene *allo*-ocimene (1.64%), also displaying a decrease in the concentrations of dillapiole (48.66–39.36%), myristicin (3.26–2.41%), and apiol (2.54–1.83%).

At 30 dpi, the hydrocarbon *n*-octane (2.43%) was identified only in the roots of inoculated plants (Table 3). Quantitatively, the production of dillapiole displayed an increase (71.65-84.65%) with a concomitant decrease in the concentrations of myristicin (17.45–10.52%) and elemicin (2.23–0.28%) (Fig. 2). In addition, there was a decrease in the amounts of α-copaene (1.20–0.30%), β-caryophyllene (1.50–0.53%), and germacrene D (1.16–0.37%).

**Table 3:** Comparison of volatile components produced in inoculated and non-inoculated roots of *P. aduncum (Mean standard deviation)*

Compound	RI^calc^	RI^lit^	30 dpi^a^		60 dpi^a^		90 dpi^a^	
			Control	AMF	Control	AMF	Control	AMF
*n*-Octane	782	800		2.43 ± 1.18		0.10 ± 0.10	0.03 ± 0.04	0.14 ± 0.04
α-Thujene	918	924			0.13 ± 0.23	0.46 ± 0.14	0.27 ± 0.37	0.33 ± 0.10
Camphene	934	946	0.39 ± 0.42	0.07 ± 0.13	1.06 ± 0.94	1.86 ± 0.45	2.77 ± 0.40	1.68 ± 0.34
Sabinene	963	969			0.05 ± 0.08	0.25 ± 0.06	0.16 ± 0.23	0.18 ± 0.31
Myrcene	973	988		0.02 ± 0.03		0.16 ± 0.14		
β-Pinene	974	974				0.07 ± 0.13	0.17 ± 0.23	0.30 ± 0.03
δ-3-Carene	998	1008	0.25 ± 0.24		0.71 ± 1.00	1.77 ± 0.46	1.20 ± 0.23	0.82 ± 0.54
*p*-Cymene	1013	1020						
Limonene	1018	1024			0.06 ± 0.10	0.22 ± 0.15	0.16 ± 0.03	0.30 ± 0.23
(*Z*)-β-Ocimene	1033	1032	0.05 ± 0.09		0.26 ± 0.29	0.58 ± 0.06	0.86 ± 0.30	0.68 ± 0.19
(*E*)-β-Ocimene	1043	1044	0.05 ± 0.09		0.08 ± 0.14	0.34 ± 0.06	0.77 ± 0.21	0.57 ± 0.24
*p*-Mentha-2,4(8)-diene	1074	1085				0.04 ± 0.04		0.05 ± 0.04
Linalool	1090	1095						0.07 ± 0.06
*allo*-Ocimene	1119	1128				0.09 ± 0.01	0.09 ± 0.13	0.79 ± 1.32
Camphor	1138	1141	0.49 ± 0.30	0.10 ± 0.10	0.43 ± 0.52	1.28 ± 0.66	1.34 ± 1.89	0.11 ± 0.03
Camphene hydrate	1147	1145			0.01 ± 0.02	0.04 ± 0.04	0.06 ± 0.08	0.09 ± 0.02
Isoborneol	1147	1155	0.12 ± 0.21		0.15 ± 0.26	0.62 ± 0.29	0.43 ± 0.60	0.39 ± 0.13
Borneol	1156	1165			0.06 ± 0.11	0.06 ± 0.05	0.06 ± 0.08	0.04 ± 0.04
Citral	1170	1174					0.04 ± 0.06	0.02 ± 0.03
Terpinen-4-ol	1172	1174						0.04 ± 0.03
α-Terpineol	1187	1186	0.02 ± 0.03		0.04 ± 0.08	0.13 ± 0.04	0.14 ± 0.00	0.16 ± 0.02
Piperitone	1246	1249			0.06 ± 0.10	0.14 ± 0.09	0.04 ± 0.06	0.08 ± 0.14
Safrole	1282	1285	0.05 ± 0.04		0.06 ± 0.05	0.10 ± 0.12	0.07 ± 0.01	0.22 ± 0.15
δ-Elemene	1333	1335	0.11 ± 0.04	0.13 ± 0.16	0.52 ± 0.26	0.36 ± 0.25	0.40 ± 0.23	0.29 ± 0.04
α-Cubebene	1342	1345			0.04 ± 0.03	0.06 ± 0.07	0.03 ± 0.04	0.02 ± 0.03
Cyclosativene	1365	1369	0.17 ± 0.14		0.42 ± 0.10	0.28 ± 0.17	0.34 ± 0.03	0.32 ± 0.03
α-Copaene	1372	1374	1.20 ± 0.51	0.30 ± 0.12	2.35 ± 0.32	1.34 ± 0.62	1.91 ± 0.22	1.63 ± 0.24
β-Cubebene	1385	1387			0.12 ± 0.11		0.27 ± 0.12	
β-Elemene	1386	1389	0.26 ± 0.19	0.02 ± 0.03	0.59 ± 0.18	0.65 ± 0.41	0.34 ± 0.18	0.58 ± 0.08
*cis*-α-Bergamotene	1411	1411			0.01 ± 0.01			
β-Caryophyllene	1416	1417	1.50 ± 0.39	0.53 ± 0.16	3.00 ± 0.09	2.35 ± 1.01	1.93 ± 0.27	2.03 ± 0.08
β-Copaene	1427	1430	0.22 ± 0.23	0.08 ± 0.08	0.99 ± 0.08	0.70 ± 0.36	0.60 ± 0.16	0.64 ± 0.05
γ-Muurolene	1428	1478	0.21 ± 0.36					
*trans*-α-Bergamotene	1430	1432	0.02 ± 0.03		0.08 ± 0.04	0.07 ± 0.08	0.03 ± 0.04	0.05 ± 0.04
Aromadendrene	1435	1439			0.03 ± 0.05	0.03 ± 0.05		
*cis*-Muurola-3,5-diene	1442	1448	0.02 ± 0.03		0.04 ± 0.06			
6,9-Guaiadiene	1443	1442				0.16 ± 0.10	0.13 ± 0.09	0.14 ± 0.02
*trans*-Muurola-3,5-diene	1445	1451	0.02 ± 0.03		0.03 ± 0.03	0.02 ± 0.03		
*allo*-Aromadendrene	1448	1458					0.05 ± 0.07	0.06 ± 0.01
α-Humulene	1452	1452	0.40 ± 0.21	0.15 ± 0.08	0.90 ± 0.38	0.83 ± 0.50	0.69 ± 0.22	0.72 ± 0.04
(*E*)-β-Farnesene	1452	1454			0.14 ± 0.24			
β-Santalene	1456	1457	0.06 ± 0.06		0.17 ± 0.09	0.18 ± 0.11	0.11 ± 0.04	0.13 ± 0.01
*trans*-Cadina-1(6),4-diene	1469	1475			0.02 ± 0.04	0.06 ± 0.05		1.34 ± 1.68
α-Neocallitropsene	1472	1474					0.08 ± 0.01	0.03 ± 0.05
γ-Curcumene	1476	1481			0.68 ± 1.12	0.05 ± 0.05		
Germacrene D	1477	1484	1.16 ± 0.58	0.37 ± 0.16	1.39 ± 1.23	1.60 ± 0.67	1.59 ± 0.48	1.64 ± 0.12
β-Selinene	1486	1489				0.05 ± 0.05		
γ-Muurolene	1488	1478			0.03 ± 0.05			
*trans*-Muurola-4(14),5-diene	1489	1493			0.05 ± 0.08	0.11 ± 0.06		0.05 ± 0.04
α-Selinene	1492	1498	0.03 ± 0.05		0.06 ± 0.10			
Bicyclogermacrene	1492	1500	0.04 ± 0.06		0.19 ± 0.22	0.42 ± 0.27	0.22 ± 0.13	0.25 ± 0.02
α-Muurolene	1495	1500	0.10 ± 0.09		0.29 ± 0.07	0.26 ± 0.13	0.20 ± 0.04	0.23 ± 0.03
Pentadecane	1499	1500	0.21 ± 0.09	0.15 ± 0.12	0.41 ± 0.07	0.23 ± 0.10	0.19 ± 0.02	0.27 ± 0.06
β-Bisabolene	1505	1505	0.02 ± 0.03		0.08 ± 0.07	0.08 ± 0.09	0.07 ± 0.02	0.13 ± 0.06
β-Curcumene	1507	1514	0.02 ± 0.03		0.06 ± 0.07	0.10 ± 0.06	0.03 ± 0.04	0.04 ± 0.03
γ-Cadinene	1510	1513			0.02 ± 0.04	0.05 ± 0.05		
Cubebol	1513	1514				0.18 ± 0.31	0.28 ± 0.39	
α-Cadinene	1515	1537	0.28 ± 0.12	0.05 ± 0.05	0.28 ± 0.48	0.39 ± 0.42		
Myristicin	1519	1517	17.45 ± 2.09	10.52 ± 2.95	18.38 ± 1.01	19.93 ± 1.13	19.25 ± 1.46	18.47 ± 0.91
(*E*)-γ-Bisabolene	1524	1529			0.05 ± 0.05	0.03 ± 0.05	0.03 ± 0.04	0.02 ± 0.03
*trans*-Cadina-1.4-diene	1529	1533			0.04 ± 0.04	0.10 ± 0.05	0.03 ± 0.04	0.05 ± 0.05
α-Cadinene	1535	1537				0.02 ± 0.03		
α-Copaen-11-ol	1539	1539	0.03 ± 0.05		0.14 ± 0.14	0.28 ± 0.09	0.16 ± 0.11	0.20 ± 0.08
Elemicin	1545	1555	2.23 ± 1.16	0.28 ± 0.36	2.22 ± 0.33	3.13 ± 0.43	3.56 ± 1.06	3.92 ± 0.75
(*E*)-Nerolidol	1559	1561	0.06 ± 0.06		0.13 ± 0.11	0.35 ± 0.22	0.17 ± 0.24	0.27 ± 0.10
Spathulenol	1573	1577				0.08 ± 0.09		
Germacrene D-4-ol	1574	1574					0.05 ± 0.07	
Caryophyllene oxide	1575	1582	0.02 ± 0.03		0.06 ± 0.05	0.19 ± 0.12	0.08 ± 0.01	0.07 ± 0.02
6-Methoxyelemicin	1579	1595	0.02 ± 0.03			0.21 ± 0.14	0.18 ± 0.25	0.35 ± 0.18
Viridiflorol	1592	1592				0.06 ± 0.10		
Humulene epoxide II	1607	1608				0.02 ± 0.03		
Dillapiole	1618	1620	71.65 ± 7.30	84.65 ± 2.66	61.12 ± 6.71	54.15 ± 6.56	54.27 ± 5.32	51.96 ± 5.88
1-*epi*-Cubenol	1627	1627			0.13 ± 0.12			
Muurola-4,10(14)-dien-1-β-ol	1636	1630				0.15 ± 0.25		
α-*epi*-Muurolol	1649	1640				0.08 ± 0.09		
α-Cadinol	1660	1652			0.01 ± 0.02	0.06 ± 0.07		0.02 ± 0.03
Apiole	1670	1677	0.64 ± 0.40	0.06 ± 0.11	0.85 ± 0.37	1.72 ± 0.68	1.59 ± 0.78	1.78 ± 0.37
Monoterpene hydrocarbons			0.74 ± 0.84	0.09 ± 0.16	2.35 ± 2.78	5.84 ± 1.70	6.45 ± 2.13	5.70 ± 3.34
Oxygenated monoterpenes			0.63 ± 0.54	0.10 ± 0.10	0.75 ± 1.09	2.27 ± 1.17	2.11 ± 2.77	0.93 ± 3.34
Sesquiterpene hydrocarbons			5.84 ± 3.18	1.63 ± 0.84	12.67 ± 5.73	10.35 ± 5.79	9.05 ± 2.51	10.41 ± 2.82
Oxygenated sesquiterpenes			0.13 ± 0.17		0.52 ± 0.48	1.56 ± 1.51	0.77 ± 0.86	0.63 ± 0.24
Phenylpropanoids			92.04 ± 11.02	95.51 ± 6.08	82.63 ± 8.47	79.24 ± 9.06	78.92 ± 8.88	76.70 ± 8.24
Others			0.21 ± 0.09	2.58 ± 1.30	0.41 ± 0.07	0.33 ± 0.20	0.22 ± 0.06	0.41 ± 0.10
Total			99.59 ± 15.84	99.91 ± 8.48	99.33 ± 18.62	99.59 ± 19.43	97.52 ± 17.21	94.78 ± 18.08

dpi: Days post inoculation; Control: *P. aduncum* non-inoculated with AMF; AMF: *P. aduncum* inoculated with AMF; RIcal: Retention index calculated; RIlit: Retention Index of Library

* Statistical difference according to Bonferroni test (*p* < 0.05)

^a^Microsoftean ± standard deviation (n = 3)

At 60 dpi, important changes were observed: the inoculated plants produced 15 compounds which were absent in the control group. Dillapiole production showed a decrease (61.12–54.15%) and a slight increase in the production of myristicin (18.38–19.93%) and elemicin (2.22–3.13%) was observed (Fig. 2). At 90 dpi, only inoculated plants produced detectible (> 0.1%) levels of the sesquiterpene *trans*-cadina-1(6),4-diene (1.34%). The phenylpropanoids myristicin (19.25–18.47%) and dillapiole (54.27–51.96%) showed a decrease as well as the sesquiterpene hydrocarbons δ-elemene (0.40–0.29%) and α-copaene (1.91–1.63%). The minor compounds produced in the roots showed a behavior different than leaves, with a decrease of monoterpenes (*Z*)-β-ocimene (0.86–0.68%) and (*E*)-β-ocimene (0.77–0.57%) in inoculated plants (Table 3).

### Evaluation of lipoxygenase activity in *P. aduncum* during AMF inoculation

**Fig. 3:**
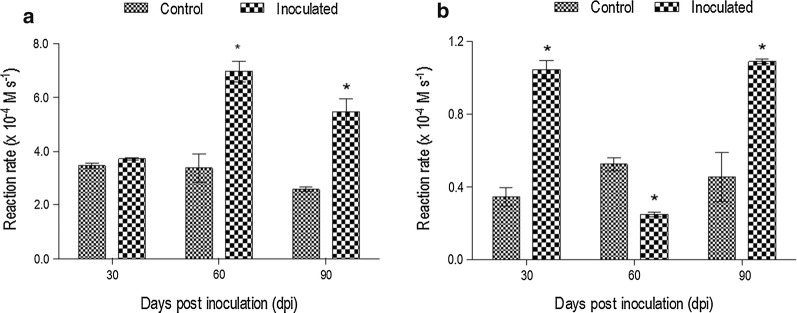
Variation of LOX activity in inoculated and non-inoculated plants of *P. aduncum*. **a** Leaves; **b** Roots. *Statistical difference according to Bonferroni test (*p* < 0.05)

LOX activity was about 4 times greater in the leaves compared to the roots of *P. aduncum*. The leaves of inoculated plants showed an increase of LOX activity at 60 and 90 dpi (Fig. 3a). However, in the roots its increase was observed only at 30 and 90 dpi to inoculated plants (Fig. 3b).

## Discussion

In the first stage of mycorrhizal colonization, the formation of the penetration apparatus (hyphopodium) occurred, presumably due to the recognition of signaling molecules of the plant by AMF, after the exchange of biochemical signals between fungus and host (Gianinazzi-Pearson and Brechenmacher 2004; Requena et al. 2002). After the formation of the appressorium in the epidermis and the intracellular extension of the hyphae, the AMF was established between the cell walls of the plant until reaching the cortex (Kiriachek et al. 2009). *P. aduncum* roots showed a typical *Arum*-type colonization, which consists of an extension of the intracellular hyphae at the beginning of colonization, followed by penetration into the cells of the root cortex, forming terminal arbuscles that bind the hyphae through arbuscular trunks (Fig. 1f) (Smith and Smith 1997). *Arum*-type colonization has also been observed in roots of 22 plant species including *Piper nigrum*, inoculated with AMF from the genus *Acaulospora, Gigaspora, Glomus* and *Scutellospora* (Muthukumar and Tamilselvi 2010).

The presence of mycorrhizal structures into radicular cells indicates the colonization and exchange of nutrients between host plants and AMF, mostly in arbuscules, which are considered the key in this process, and present a development cycle until degeneration. In addition, water is absorbed by the external mycelium and moves through the hyphae, which favors the apoplastic flow in the root system of the plant (Bárzan et al. 2012). The vesicles are globular or elliptical structures, which store lipids and glycogen, serving as a reserve organ for the fungus, and their formation can occur within or between the cells of the root cortex (Smith and Read 2008). These fungal structures were also observed in roots of plants of *Poincianella pyramidalis* and *Cnidoscolus quercifolius* that were inoculated by *Acaulospora longula* and *C. etunicatum* (Frosi et al. 2016).

The developmental parameters of inoculated plants showed significant variation only in the height of plants at 30 and 90 dpi and node number at 90 dpi. Our results are distinct in comparison with *Piper longum* plants inoculated with *Glomus fasciculatum, Acaulospora foveata* and *Gigaspora margarita*, which showed an increase in leaf number. However, there was a decrease in root development, mostly for plants inoculated with *G. fasciculatum* (Seema and Garampalli 2015). The height variation in *P. aduncum* plants was similar to that observed in basil (*Ocimum basilicum*) and rosemary (*Rosmarinus officinalis*) inoculated with *G. clarum* spores, which showed an increase of 45.49 and 25.93%, respectively (Russomanno et al. 2008). AMF contributes to increasing photosynthesis rate, favoring plant growth (Tanaka and Fujita 1979). The increase in height, but not in the number of leaves, indicates a possible production of photoassimilates directed to the needs of the plant (Neumann et al. 2009). The AMF species *Gigaspora margarita*, *Acaulospora longula* and *C. etunicatum* were considered as promoters of growth and better biomass production in *P. longum* seedlings (Seema and Garampalli 2015).

The contribution of AMF to increases of nutrients and biomass can be important when nutrient availability in the soil is low, thereby promoting a higher efficiency through the benefits of photoassimilates produced in the host plant (Neumann et al. 2009; Smith and Smith 1997; Xie et al. 2014). In this case, this hypothesis can explain the lower variation in the biomass production in *P. aduncum* because we have used a soil rich in nutrients. The production of substances by inoculated plants may be related to defense mechanisms during the AMF colonization, which led to increased expression of defense-related genes and production of volatile compounds such as alcohols, ethers, and aldehydes in their leaves (Quaglia et al. 2012). These metabolites are produced by enzymes, including the lipoxygenases, and are considered compounds involved in signaling and defense (Liavonchanka and Feussner 2006).

Several chemical compounds are involved in the plant interaction, including low-molecular-weight and monoterpenes such as myrcene and mixtures of ocimene isomers made up of (*E*)-β-ocimene, *(Z*)-β-ocimene and *allo*-ocimene (Godard et al. 2008). Among the minor compounds, (*E*)-β-ocimene and (*Z*)-β-ocimene showed a gradual increase during inoculation by AMF in the leaves and a decrease in the roots. These compounds are emitted by plants to response to herbivore attack and changes in abiotic factors (Gouinguené and Turlings 2002).

Our observations showed a correlation with LOX activity in the leaves of inoculated plants; the increase of LOX activity indicating a possible defense mechanism of plant (Baysal and Demirdoven 2007). The regulation of the by-product of the LOX pathway, jasmonic acid, can promote changes in the colonization level by AMF in plants (Gutjahr et al. 2015). The activation of the LOX pathway enhances important functions in the primary and secondary metabolism in the plants (Morcillo et al. 2012).

LOX is involved in the production of volatile compounds in leaves and roots such as alcohols, ethers, and aldehydes, which are considered both signaling and defense compounds (Liavonchanka and Feussner 2006). After biotic and abiotic stress, LOX activity is increased, and it depends mostly on inducing agents as well as the plant genotype and physiologic conditions (Silva et al. 2004). The activation of LOX activity was induced by inoculation in the roots of *Rhizophagus irregulars* in bean plants (*Phaseolus vulgaris* L.) (Mora-Romero et al. 2015). At 21 dpi, LOX activity was increased about 50% in *P. divaricatum* seedlings infected by *Fusarium solani* f. sp. *piperis* (Meireles et al. 2016).

*P*. *aduncum* presents many biological activities reported in the literature, which are attributed to the phenylpropanoid dillapiole (Bernard et al. 1995; Almeida et al. 2009; Souto et al. 2012). Alternatives to increase the production of dillapiole were investigated through the inoculation by AMF in the roots. The results showed dillapiole production decreased in roots and leaves. However, several monoterpenes and sesquiterpenes increased in the leaves, and 15 components were produced in the roots of inoculated plants. These results indicate a metabolic activity was induced by the inoculation of AMF and can serve to contribute to future studies on plant-fungal interactions.
